# Acute Renal Failure Due to Bile Cast Nephropathy: An Overlooked Cause of Kidney Injury

**DOI:** 10.7759/cureus.9724

**Published:** 2020-08-13

**Authors:** Muhammad B Jamshaid, Phool Iqbal, Aamir Shahzad, Zohaib Yousaf, Mohamed Mohamedali

**Affiliations:** 1 Internal Medicine, Hamad General Hospital, Doha, QAT; 2 Medicine, Hamad General Hospital, Doha, QAT; 3 Internal Medicine, Hamad Medical Corporation, Doha, QAT; 4 Clinical Research, Dresden International University, Dresden, DEU; 5 Medicine, Hamad Medical Corporation, Doha, QAT

**Keywords:** obstructive jaundice, acute kidney injury, bile cast nephropathy

## Abstract

Acute kidney injury in the setting of hyperbilirubinemia presents a diagnostic challenge. Hepatorenal syndrome takes precedence as a diagnosis in these cases. Bile cast nephropathy is a diagnosis that gets relatively low consideration. The most accurate diagnostic tool for bile cast nephropathy is a renal biopsy, which may present a challenge in certain clinical settings. There are no set guidelines for its management. While the exact cause of the condition is unknown, it is presumed to be secondary to multiple concurrent insults to the kidney including direct toxicity from bile acids, obstruction caused by bile casts, and systemic hypo-perfusion from vasodilation. It is believed that plasmapheresis and albumin dialysis have been associated with some recovery of renal function. We present a case of acute renal failure in a patient with obstructive jaundice, who responded to dialysis and biliary drain insertion.

## Introduction

Bile cast nephropathy is characterized by deterioration of renal function in the presence of hyperbilirubinemia. High levels of bilirubin (>400 umol/l) can lead to acute kidney injury through multiple mechanisms, i.e., due to the direct toxic effect of bilirubin on renal tubular cells and the obstruction of nephron with bile cast; the diagnosis is often challenging since patients usually have impaired coagulation profile at the time presentation, which makes kidney biopsy nearly impossible to perform. But certain clues like bile crystals and granular case on urinalysis can help in the diagnosis of bile cast nephropathy. Management guidelines do not exist since only a few cases have been reported so far [1-5]. Therapies aimed at decreasing bilirubin such as percutaneous biliary drainage (as in our case), albumin dialysis, and plasmapheresis have been used in the management with varied success [2]. We report a case of obstructive jaundice treated presumptively as bile cast nephropathy, which responded to biliary drainage.

## Case presentation

A 64-year-old male with multiple comorbidities, including diabetes mellitus, hypertension, chronic liver disease, and chronic kidney disease stage III, presented with yellow sclerae and dark urine. The examination was unremarkable except for scleral icterus and excoriations secondary to itching. Blood workup showed unremarkable cell count with predominantly direct hyperbilirubinemia. Imaging revealed dilated intrahepatic biliary radicles, common bile duct, and a pancreatic head lesion. Biopsy of the head of the pancreas revealed adenocarcinoma not amenable to surgery due to the high perioperative risk [American Society of Anesthesiologists (ASA) class 4]. The decision to proceed with endoscopic retrograde cholangiopancreatography (ERCP) and stenting was made while seeking a second opinion from a high-volume center.

**Figure 1:**
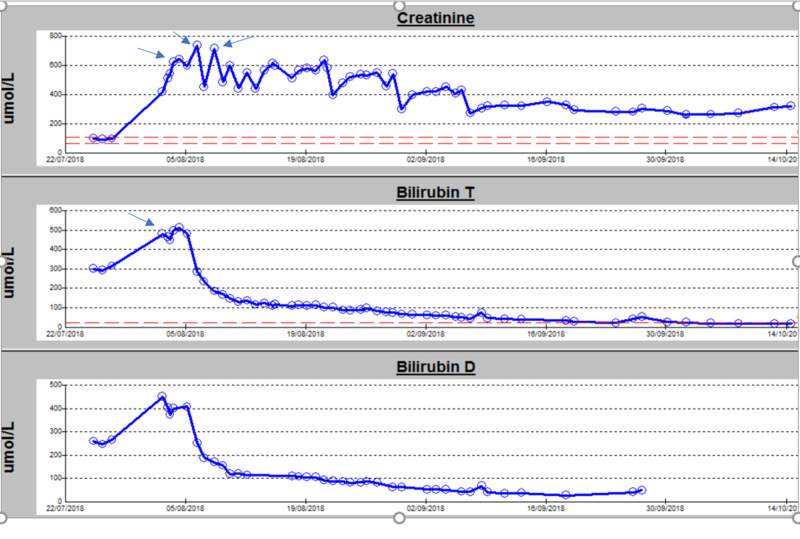
Variations in bilirubin and renal parameters during the hospital stay Arrows in creatinine graph denote dialysis sessions, and arrow in total bilirubin graph denotes the insertion of the biliary drain Bilirubin T: total bilirubin; Bilirubin D: direct bilirubin

On hospital day four, before undergoing ERCP, the patient developed hypotension and features suggestive of acute cholangitis with raised inflammatory markers [white blood count (WBC): 17,000 per microliter; C-reactive protein (CRP): 130 mg per liter; procalcitonin: 3.2 nanograms per milliliter]. The patient developed anuria and acute renal failure. Hypotension was fluid-responsive, but he remained anuric. He was initiated on antibiotics, and a biliary drain was inserted to remove bilirubin from the body and avoid further kidney damage. Hemodialysis was initiated (as renal replacement therapy) with a plan to proceed to plasma exchange in case of inadequate response. After three sessions of hemodialysis, the patient started to produce urine, and his renal function improved, as demonstrated in Figure 1 and Table 1, respectively. The patient did not require any additional renal replacement therapy, and he was discharged home without any further complications.

**Table 1:** Investigations on admission, pre-dialysis, and post-dialysis PTC: percutaneous transhepatic cholangiography; ALT: alanine aminotransferase; AST: aspartate aminotransferase; ALP: alkaline phosphatase

Variables	Normal values	On admission	Pre-dialysis	Post-dialysis/after PTC
Urea	2.8-8.1 mmol/l	18.8 mmol/l	25.90 mmol/l	20.3 mmol/l
Creatinine	62-106 µmol/l	168 µmol/l	499 µmol/l	319 µmol/l
Total bilirubin	0-21 µmol/l	330 µmol/l	513 µmol/l	11 µmol/l
Direct bilirubin	1.71-20.5 µmol/L	274 µmol/l	409 µmol/l	---
ALT	0-41 u/l	295 u/l	614 u/l	12 u/l
AST	0-40 u/l	171 u/l	982 u/l	17 u/l
ALP	40-129 u/l	520 u/l	1,250 u/l	196 u/l

## Discussion

Cholemic nephrosis was first described in 1899 and heralds the presence of renal injury secondary to components of bile [1]. Bile cast nephropathy is a type of acute kidney injury that occurs in various circumstances like obstructive jaundice, hepatorenal syndrome, drug-induced severe cholestatic liver injury, non-immune hydrops fetalis-associated jaundice, infectious mononucleosis-associated jaundice, and renal failure, hemolytic jaundice and cirrhosis, and fulminant hepatitis [2]. However, this entity is often overlooked and has been reported rarely in the literature [3,4]. A clinicopathologic study from the University of Chicago involving 44 subjects with jaundice and renal injury showed that 24 patients had bile casts upon biopsy/autopsy, with extension to distal nephron segments in 18 mild cases, and six of them had severe involvement up to the level of proximal tubules [3].

In the case of our patient, we considered a differential diagnosis of septic shock secondary to cholangitis leading to acute kidney injury, hepatorenal syndrome, and bile cast nephropathy. The patient did not respond to fluid resuscitation and broad-spectrum antibiotics; hence, septic shock was ruled out as a cause of acute renal injury. Moreover, treatment with albumin and terlipressin for the suspected hepatorenal syndrome was also futile. The patient's renal function continued to deteriorate, and he remained anuric. Renal replacement therapy was initiated as the patient went into volume overload and did not respond to medical management. After biliary drain insertion and three sessions of renal replacement therapy, his direct bilirubin levels started to decline, and the patient started to produce urine as well. The diagnosis of bile cast nephropathy was made after concomitant improvement of renal function and normalization of bilirubin levels. A kidney biopsy is needed to confirm the diagnosis of bile cast nephropathy, with typical histological features of granular casts and pigmentation in renal tubules due to bile visualized with Hall’s special stain [6]. However, our patient's condition improved, and kidney biopsy was not performed due to the high risk associated with it.

## Conclusions

Acute kidney injury in the setting of hyperbilirubinemia is usually attributed to hepatorenal syndrome; however, there are alternative diagnostic considerations such as bile cast nephropathy, which is an under-reported entity. Bile cast nephropathy should be considered when encountering a patient with kidney injury in the setting of hyperbilirubinemia. A timely diagnosis of the condition and prompt initiation of therapy can lead to a favorable clinical response.
